# Teaching primary care teamwork: a conceptual model of primary care team performance

**DOI:** 10.1111/tct.13037

**Published:** 2019-07-07

**Authors:** Carole M Warde, Karleen F Giannitrapani, Marjorie L Pearson

**Affiliations:** ^1^ Center of Excellence Primary Care Education VA Greater Los Angeles Healthcare System; ^2^ University of California Los Angeles David Geffen School of Medicine; ^3^ VA HSR&D Center for Innovation to Implementation; VA Palo Alto Health Care System; ^4^ Primary Care and Population Health Stanford University School of Medicine Palo Alto; ^5^ Health RAND Corporation Santa Monica CA

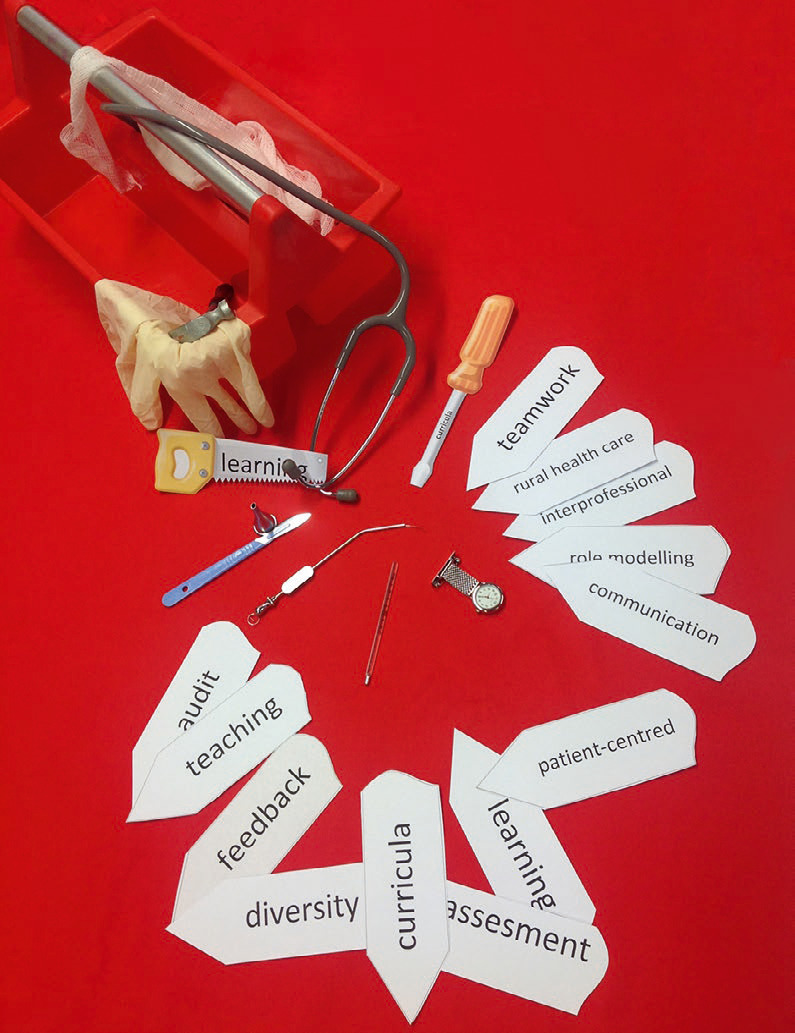

## Introduction

Over the past two decades, team‐based care models have advanced the delivery of primary care.^1^ Primary care teams consist of two or more professionals and staff who share the goal of delivering high‐quality, patient‐centred care to patients and their families through collaboration and coordination.^2^ In general, teamwork requires each member to have role‐specific competence, a shared structure to work within, and ‘coordinative and adaptive capabilities’.^3^ Their ability to work together depends on their collective development: team training enhances team performance.^4^ Teams that lack common goals, leadership or training experiences present a particularly complex undertaking.^2^, ^3^

… teamwork requires each member to have role‐specific competence, a shared structure to work within, and “coordinative and adaptive capabilities”

Over the last 45 years, the need for interprofessional team‐based training for health professions has been well documented.^5^ Effective team training is tailored to the specific tasks, behaviours and relationships of a health care team.^5^ Although there are important examples of team training in health care, they do not address the specific characteristics of primary care teamwork in the context of existing resources and culture.^5^

Much has been learned about primary care teamwork from the patient‐centred medical home (PCMH), a team‐based one‐care model implemented extensively in the USA. This approach honours the tenets of primary care – comprehensive, coordinated, accessible and continuous – as well as emphasising the importance of the personal relationship between a patient and the care team members.^6^ The PCMH has led to improvements in patient experience, care quality, cost and team member well‐being.^1^ Several group practices have been shown to promote team performance: goal specification, a shared understanding of team roles, quality improvement (QI) skills, team coordination, relationship‐centred communication and adequate meeting time.^7^, ^8^, ^9^, ^10^

A straightforward framework to learn, assess and improve primary care teamwork would be a valuable tool for medical professionals and trainees to learn team‐related competencies. In this article, we describe a conceptual model that integrates principles of primary care team performance with research on what constitutes effective teamwork. We suggest strategies for how learners, faculty members and other stakeholders can use the model to enhance primary care team practice, training and assessment.

## Approach

In 2010, the Veterans Administration (VA; provides near‐comprehensive health care services to eligible military veterans) adopted the PCMH care model in 900 primary care clinics. We developed the Conceptual Model of Primary Care Team Performance (CM of PCTP) at a VA demonstration site for PCMH implementation. Our workgroup includes two VA clinician‐educators with team‐based practice expertise in patient‐centred communication and social scientists with experience in teamwork, quality improvement, coaching, and innovation development and assessment.

We selected the evidence‐based ‘Integrative Framework of Team Effectiveness’ as our starting point because of its validity and flexibility to include primary care practice characteristics.^11^ This framework is a synthesis of 138 models of teamwork from organisational development experts. It includes the four traditional constructs of teamwork: inputs, team performance, outputs and culture. Developers of this framework intended it to be adapted to many fields, and to serve as a foundation for team performance evaluation, the testing of new hypotheses and designing effective team training interventions.

## The Conceptual Model of Primary Care Team Performance

**Figure 1:**
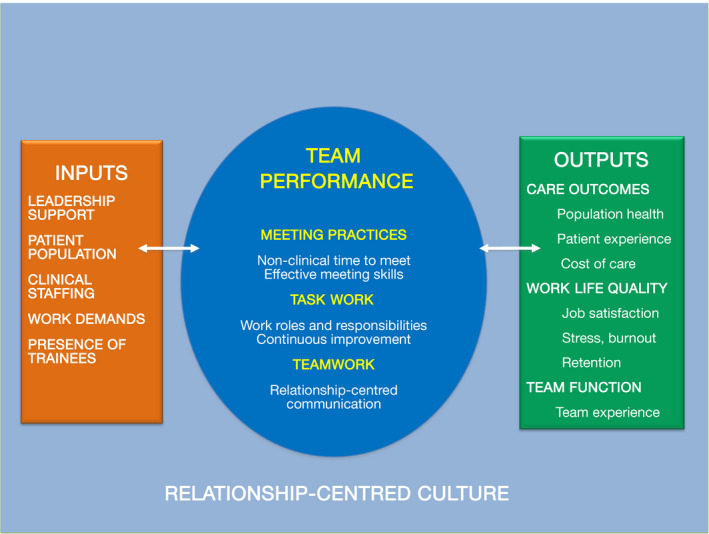
Conceptual model of primary care team performance.

As shown in Figure 1, our model combines the key constructs of teamwork with characteristics relevant to a team‐based primary care practice. Each aspect of this model contributes to what helps a team work well together, and requires a different set of knowledge and skills.

Team performance in the central oval represents the essential processes that occur as a team works to coordinate patient care (Figure 1). It incorporates three related domains of team performance with effective aspects of primary care such as patient–team partnership, data‐driven improvement, planned opportunities for team communication and population management.^5^, ^9^

○ Meeting practices – routine meetings are scheduled during non‐clinical time and the team uses processes that promote efficient and meaningful care coordination and improvement (Table 1). Although difficult to arrange in a busy clinical practice, teams must have uninterrupted time and space to build relationships, reflect on how they work together, and coordinate care.^9^, ^12^, ^13^ Team meetings and huddles are the most common formats used by primary care teams to coordinate their work.
  **Table 1:** Teaching meeting practices: competencies and learning strategies^9^, ^12^, ^13^

**Competencies:** ○ Regularly meet using sound practices ○ Attend all planned meetings of the team ○ Value protected meeting time ○ Use effective meeting practices	
**Learning strategies:**	
1 Clarify meeting aims	Before the meeting, a proposed agenda with expected outcomes is prepared and distributed, with consideration of useful meeting processes
Volunteer for meeting roles	Meeting leader: focus on agenda, manage time, facilitate participation and discussion, guide the team to build consensus and reflect on process Facilitator: help the meeting leader by monitoring meeting focus, flow and social processes, reminding others of ground rules, and helping when things get stuck Recorder: document progress, decisions, next steps, items for later discussion, and prepare and distribute minutes Timekeeper: track the time for each item and alert others before the time is up
Follow ground rules	The team agrees upon expected team member behaviours
Negotiate agenda and time for each item	Review agenda, place most important items first, be realistic about time
Complete the agenda	Discuss information, make decisions, plan next steps
Review actions	Summarise accomplishments, items for follow‐up and responsible people
Set next meeting aims	Plan a preliminary agenda for the next meeting
Reflect on the meeting process	What worked? How well did we discuss the information? How well did we respond to each other's questions? What needs to be improved?
○ Task work is the delivery of high‐quality patient‐centred care to a specific patient population (Table 2). Care is contingent on team members knowing and performing their own roles and responsibilities *and* understanding those of team members.^7^, ^8^ Also important is their commitment and ability to meet patients’ needs and to continuously learn and improve work processes and care outcomes.^2^, ^12^
○ Teamwork describes the communication processes required for collaborative work (Table 3). How team members treat each other determines whether a team works together successfully.^6^, ^12^, ^14^ Team members that communicate respectfully and develop trusted relationships will cultivate knowledge sharing, aiding team members to acclimate to each other's actions and ultimately to provide coordinated care. We have identified nine specific relationship‐centred communication skills that can be learned to promote a collaborative culture.^2^, ^12^, ^13^, ^14^, ^15^
  **Table 2:** Teaching task work: competencies and learning strategies^7^, ^8^, ^12^

**Task work competencies:** ○ Know, perform, and improve roles and responsibilities ○ Verbalise team roles (both your own and those of team members) ○ Perform your professional responsibilities, and state when y u cannot do this ○ Accept the responsibility to improve care	
**Learning strategies:**	
1 Team roles	The team can collaboratively prepare a checklist of clinical roles and responsibilities for each team member New team members or trainees can shadow other team members to understand their work roles Periodically reflect on how the team is working together Discuss ways to assist each other during busy clinic days
Perform professional responsibilities	Huddle: each day, the team can meet for 10–20 minutes to identify patient needs, to schedule changes, and to coordinate care Case conferences: periodic meetings with the whole team to reflect on challenging cases and to identify ways to coordinate care and/or learn new knowledge or practices Panel management sessions: with relevant team members, reflect on a registry of one care outcome for the team's patient panel (i.e. Haemoglobin A1c) and initiate plans to improve
Improve care	A workshop series to learn and apply the basic principles of quality improvement (QI) Identify team improvement needs related to measures of work processes or care measures Team meetings can provide a set time to identify and work on QI projects Promote a culture of safety by accepting mistakes and admissions of not knowing as opportunities to learn and improve

**Table 3:** Teaching teamwork: competencies and learning strategies^2^, ^12^, ^15^

**Teamwork competencies:** ○ Relationship‐centred communication behaviours ○ Value patient‐centred care ○ Respect culture and values of team members ○ Act with honesty and integrity ○ Make collaborative decisions ○ Skillfully engage in difficult conversations ○ Hold team members accountable ○ Manage conflict respectfully ○ Seek and give feedback	
**Learning strategies:**	
Listen actively	Listen without interruption or judgement Elicit others’ perspectives Reflect back understanding One person speaks at a time
Build trust	Disclose feelings, weaknesses and relevant experiences
Hold team members accountable	Respond to the inappropriate remarks made by others Address misunderstandings Follow up on the ‘to do’ list Discuss agreed‐upon duties
Make decisions by consensus	Participate openly in discussions Encourage different opinions Suggest solutions Understand and explain final decisions Final decisions are a synthesis of ideas, not a compromise
Manage conflict	State and validate emotions Listen to others’ perspectives when opinions differ State needs Seek a mutually agreed‐upon solution
Give constructive feedback	Expect feedback Describe behaviours and effect of actions on others Suggest constructive changes Actively listen to both or all parties
Receive constructive feedback	Appreciate feedback Feel safe and supported in the feedback process Use as an opportunity to learn or improve
Manage emotions	Elicit, name, respond, understand and support the emotions of others
Support change in others	Help others to see change positively Encourage motivation and confidence for new behaviours Elicit pros and cons of options

Inputs (left side of the model) are the structural features of a team‐based care model determined by practice leadership. Patient demographics define the types, roles and staffing needed on the team, as well as expected outputs of team performance. Work demands are influenced by a team's panel size, patient complexity, and level of clinical staffing.^1^, ^5^ Health professions trainees are frequently integrated into the teams; they can contribute to patient care, but may also place extra demands on team members.

Outputs (right side of the model) are measurable outcomes that drive team performance and serve as benchmarks to monitor a team's care and improvement efforts. Clinical teams must be aware of and work towards relevant goals and outcomes. Patient experience, population health and cost are widely accepted as important aims of health system performance.^16^ Successful teamwork also contributes to individual and team viability and happiness, as reflected in measures of job satisfaction, stress, burnout and retention.^17^ Team perceptions of their own cohesion can provide valuable feedback on how they are working together.^18^

A relationship‐centred culture (bottom of the model) describes the optimal work environment to promote team performance. Important contributors to culture are leadership style, performance mandates, work demands, staffing, autonomy and acceptable patterns of communication.^8^, ^17^ Patient‐centredness, psychological safety, a mindset for change and reflective capacity are characteristics of a relationship‐centred culture.^5^ These play an important role in the satisfaction, well‐being, and learning ability of teams and individuals.^19^

A straightforward framework to learn, assess and improve primary care teamworkwould be a valuable tool for medical professionals … to learn team‐related competencies

## Team Performance Teaching Strategies

We propose that the CM of PCTP can be easily recalled and applied in three categories: *meeting practices* – team members coordinate their work during effective meetings; *task work* – team members value patient‐centred care, perform their roles and strive for continuous improvement; and *teamwork –* team members communicate with honesty and respect, and value relationships with team members. Both new and existing clinical teams can use this simple three‐part framework of team performance to stimulate the quick recall of what constitutes team performance, and as a starting point to assess and improve their own team performance.

The Conceptual Model of Primary Care Team Performance combines key constructs ofteamwork with characteristics of a primary care practice

Teaching strategies for teams seeking to initiate or improve team‐based education are described in Tables 1 to 3. We propose that the best way for new trainees to learn to practice in teams is to be immersed in a team and to be supported as they learn to practice their professional roles, with workshops to address new skills. Planning for trainees to join a care team is essential and lays the groundwork for effective learning for the whole team (Table 4). The very process of preparing for new learners will inevitably lead to better team‐based patient care as team members reflect on their roles, and how and where they work and meet together. Role modelling, observation and feedback by faculty members and team members are key teaching strategies, as is continuing reflection of the care and processes of teamwork.

**Table 4:** Best practices to prepare trainees for team performance training

Prior to team training:
Get a commitment from clinic leaders, team clinicians and staff to take on the role of individual and team faculty members Arrange the trainee schedules to practice as a member of an existing, well‐functioning team Consider space and time for team members to work and teach trainees Consider staffing ratios of professional faculty members to trainees to allow time for faculty members to teach Build time into the weekly schedule for huddles, case conferences, team meetings and panel management sessions Train faculty members in the skills and attitudes of good role models and coaches Plan a session for new team members and trainees to welcome them wholeheartedly, review team roles and introduce them to the team competencies as a prelude for future learning

The CM of PCTP will also assist other primary care stakeholders to learn about primary care team performance. Team members and coaches guiding teams to improve their performance can refer to the model to target areas of success and challenge. The model will provide educators with a framework to plan team training curricula and interventions. Evaluators can use the model as a roadmap to develop assessments of care outcomes, team performance, training interventions, and team member well‐being. Clinical leaders may find this framework helpful to analyse the effects of changes in the structure of a primary care practice (i.e. team membership, staffing and panel size) on team performance and care outcomes. Finally, all stakeholders can promote team performance and a relationship‐centred culture by clearly defining roles, communicating honestly and respectfully, and by planning for effective meetings.

## Conclusions

In summary, the CM of PCTP acknowledges the complexities of primary care team performance, yet suggests a simplified approach to its components, implementation, evaluation and potential as an improvement tool. We intend it to be used in three ways: (1) as a tool for primary care team members and trainees to readily remember, learn and practice key characteristics of team performance; (2) as a framework for the development of coaching and training interventions for primary care teams; and (3) as a guiding structure to assess and improve a primary care team's performance, care outcomes and team member well‐being.

… teams must have uninterrupted time and space to build relationships, reflect on howthey work together, and coordinate care
